# Lactobacilli Modulate Hypoxia-Inducible Factor (HIF)-1 Regulatory Pathway in Triple Negative Breast Cancer Cell Line 

**DOI:** 10.22074/cellj.2016.4319

**Published:** 2016-05-30

**Authors:** Ali Esfandiary, Zahra Taherian-Esfahani, Atieh Abedin-Do, Reza Mirfakhraie, Mahdieh Shirzad, Soudeh Ghafouri-Fard, Elahe Motevaseli

**Affiliations:** 1 Department of Medical Genetics, Shahid Beheshti University of Medical Sciences, Tehran, Iran; 2 Department of Microbiology, School of Biology and Center of Excellence in Phylogeny Living Organisms, College of Science, University of Tehran, Tehran, Iran; 3 Department of Molecular Medicine, School of Advanced Technologies in Medicine, Tehran University of Medical Sciences, Tehran, Iran

**Keywords:** Breast Cancer, *Lactobacillus*, HIF

## Abstract

**Objective:**

Hypoxia-Inducible Factor (HIF)-1 plays an essential role in the body’s response to low oxygen concentrations and regulates expression of several genes implicated in homeostasis, vascularization, anaerobic metabolism as well as immunological responses. Increased levels of HIF-1α are associated with increased proliferation and more aggressive breast tumor development. Lactobacilli have been shown to exert anti-cancer effects on several malignancies including breast cancer. However, the exact mechanism of such effect is not clear yet. The aim of this study was to analyze the expression of selected genes from HIF pathway in a triple negative breast cancer cell line (expressing no estrogen and progesterone receptors as well as HER-2/Neu), MDA-MB-231, following treatment with two lactobacilli culture supernatants.

**Materials and Methods:**

In this experimental study, we analyzed the expression of *HIF-1α, SLC2A1, VHL, HSP90, XBP1* and *SHARP1* genes from HIF pathway in MDA-MB-231 cells, before and after treatment with *Lactobacillus crispatus* and *Lactobacillus rhamnosus* culture supernatants (LCS and LRS, respectively) by means of quantitative reverse-transcription polymerase chain reaction (qRT-PCR).

**Results:**

Both LRS and LCS had cytotoxic effects on MDA-MB-231 cells, while the former type was more cytotoxic. LRS dramatically down-regulated expression levels of the *HIF-1α, HSP90* and *SLC2A1* in the MDA-MB-231 cells. LCS had similar effect on the expression of HSP90, to what was observed in the LRS treatment. The expression level of tumor suppressor genes *VHL* and *SHARP1* were also decreased in LCS treated cells.

**Conclusion:**

Although both LCS and LRS had cytotoxic effects on the MDA-MB-231 cells, it is proposed that LRS could be more appropriate for pathway directed treatment modalities, as it did not decrease expression of tumor suppressor genes involved in HIF pathway. Down-regulation of HIF pathway mediated oncogenes by LRS suggests that the cytotoxic effects of this *Lactobacillus* may at least be partly caused by this mechanism. As previous studies have shown that inhibition of HIF-1α and HSP90 expressions have therapeutic impact on cancer treatment, the inhibitory effect of LRS on expression of these genes implies that this *Lactobacillus* can be used in treatment strategies.

## Introduction

Hypoxia-Inducible Factor (HIF)-1 plays an essential role in the body’s response to low oxygen concentrations and increases vascularization in hypoxic regions such as localized ischemia and tumors. As a transcription factor, it regulates expression of several genes implicated in homeostasis, vascularization, anaerobic metabolism as well as immunological responses. Such crucial roles indicate its opposite therapeutic potentials in ischemic and cancer patients while the latter is focus of our research. The inhibition of *HIF-1* transcriptional activity via small molecules as well as gene therapy have been proposed as effective approaches for cancer treatment (1). 

It has been shown that the level of HIF-1α in breast tumors is associated with the pathological stages, in a way that is increased in poorly differentiated lesions than in the corresponding type of well-differentiated lesions. Increased levels of HIF-1α led to higher proliferation rate, estrogen receptor (ER) and vascular endothelial growth factor (VEGF) expressions as well as forming more aggressive tumors (2). 

Regarding the previously demonstrated role of lactobacilli to exert cytotoxic effects on cancer cells via different mechanisms (3), we hypothesized that HIF mediated signaling would be a potential target for such effects of lactobacilli. Therefore, we analyzed expression of the selected genes from HIF pathway in a triple negative breast cancer (TNBC) cell line, MDA-MB-231, which was treated with two lactobacilli culture supernatants with a demonstrated anti-cancer effects (3,5). It has been shown that MDA-MB-231 is an invasive breast cancer cell line (6) which does not express ER and progesterone receptor (PR), and does not have HER-2/Neu amplification (7). The inhibitory effect of *Lactobacillus crispatus (L. crispatus)* on breast cancer cell proliferation has previously been revealed (3). In addition, *Lactobacillus rhamnosus GG (LGG)* has been shown to exert an inhibitory effect on proliferation of gastric and colon cancer cell lines (8). This *lactobacillus* strain could reduce cell cycle progression of cervical epithelial cells and cause G1 phase accumulation of the host cells (9). Furthermore, LGG has caused tumor regression in mice bearing orthotopic bladder malignancy (10). However, its effects on breast cancer proliferation have not been elucidated yet. 

We selected *HIF-1α, SLC2A1, VHL, HSP90, XBP1* and *SHARP1* genes from HIF pathway to analyze their expression, following treatment with supernatants from the mentioned lactobacilli cultures. The genes were selected, regarding their critical role in the tumorigenicity and progression of TNBC, as documented for *XBP1, HIF1* and *SLC2A1* (11,13), as well as their essential roles in stability of HIF-1 in hypoxia conditions particularly in TNBC, as demonstrated for *HSP90* (14). Finally, *VHL* and *SHARP1* have been selected as two putative tumor suppressor genes in breast cancer with indicated negative effects on the expression of *HIF* (15). 

HIF-1 is a heterodimer protein composed of HIF-1α and HIF-1β subunits. The former subunit is regulated by the O^2^ pressure, while HIF-1β is constitutively expressed (16). In addition, HIF1α have been shown to be hyper-activated in TNBCs (12). So, we just analyzed the expression of HIF-1α subunit. HIF-1 induces the expression of hundreds of target genes in hypoxic stromal and cancer cells. Increased levels of HIF-1α protein in the primary tumor biopsy has been shown to be associated with increased mortality range in several cancers, including breast cancer. In addition, higher levels of HIF-1α in the diagnostic biopsy of breast cancer patients have been associated with increased metastasis and mortality rate, even in lymph node-negative patients (16). 

*SLC2A1* encodes a transporter protein, contributing in carriage of glucose across the plasma membrane, which is the first rate-limiting step for glucose metabolism. Increased expression level of this gene has been demonstrated in nearly all human malignant cell types including breast cancer. In addition, it has been demonstrated that up-regulation of *SLC2A1* expression is associated with higher grade and proliferative index, while the rate of differentiation is reduced (17). 

XBP1 is the other protein in HIF pathway promoting TNBC tumorigenicity, by assembling a transcriptional complex with HIF-1α, to regulate the expression of HIF1-α targets. XBP1 cooperation with HIF-1α has been shown to protract a transcriptional program supporting neo-angiogenesis and cancer stem cell (CSC) maintenance (11). 

SHARP1 has a role in HIF-1α degradation through proteasome-dependent as well as ubiquitinand oxygen-independent routes. This protein can consequently prevent the expression of HIF target genes and neutralize the HIF-dependent invasive and metastatic activities in TNBC (18). 

The von Hippel-Lindau (*VHL*) is a putative tumor suppressor gene which has been proposed to exert inhibitory effects on angiogenesis and tumor cell migration, through negative regulation of HIF1 and the stromal-derived factor-1 (SDF-1) receptor, CXCR4. *In vitro* studies have demonstrated that VHL has inhibitory effects on the invasive and migratory ability of breast cancer cells (15). 

The molecular chaperone heat shock protein 90 (Hsp90) has also been shown to be a major regulator, diminishing HIF-1α transcriptional activity in a VHL-independent manner (19). In addition, it has been demonstrated that Hsp-90 is up-regulated in several types of malignancy, including breast cancer (20). 

## Materials and Methods

### Cell culture

This study has been approved by the Ethical Committee of Shahid Beheshti University of Medical Sciences (Tehran, Iran). For this experimental study, human breast cancer (MDA-MB-231) as well as human lung fibroblast (MRC5) cell lines were purchased from the Pasteur Institute, National Cell Bank of Iran. Cell culture medium was comprised of Roswell Park Memorial Institute (RPMI) 1640 medium plus 10% heat inactivated fetal calf serum, 1.5% 4-(2-hydroxyethyl)-1-piperazineethanesulfonic acid (HEPES) and 1% penicillin/streptomycin (all from Invitrogen, Carlsbad, CA, USA). The cells were maintained as monolayer cultures at 37˚C in a humidified 5% CO_2_ atmosphere for 24 hours to attach onto the dish, followed by treatments. 

### Preparation of supernatants from lactobacillus cultures

Microaerophilic conditions were used for the culture of L. crispatus strain SJ-3C-US and L. rhamnosus strain GG in de Man Rogosa Sharpe (MRS) broth (Merck, Germany, pH=6.5) at 37˚C for 24 hours. Overnight bacterial cultures had 2×10^9^c.f.u./ml. Afterward, these cultures were centrifuged at 1100 g for 15 minutes at 4˚C. The lactoba cilli supernatants (LS) were filtered through a 0.2 mm membrane filter to get rid of residual bacteria and debris. As preparing LS, the pH of the MRS broth was decreased from 6.5 to 4.05 for L. rhamnosus and 6.5 to 4.3 for L. crispatus. The lactate concentration in LS was checked using a Lactate Randox kit (Randox Laboratories, UK) according to manufacturer’s instruction. The experiments included L. crispatus supernatant at pH=4.3 (LCS); L. rhamnosus supernatant at pH=4.05 (LRS); MRS at pH=6.5 and MRS adjusted with lactate (MRL) at pH=4.05 or 4.3 (according to the corresponding lactobacilli supernatant pH). 

### MTT assay

MTT assay kit (Sigma, St. Louis, MO, USA) was used for cell growth inhibition measurement. Each well had 10^4^ cells seeded in 100 ml standard medium. After overnight incubation, 1, 2, 5, 10, 15, 20, 40, 60, 80 and 100% (v/v) of lactobacilli culture supernatants were added to MDA-MB-231 and MRC5 cells, respectively. Plates were incubated at 37˚C under 5% (v/v) CO_2_ concentration. Cell viability was calculated using the following equation: 

$\mathrm{Viability (percentage of the control)}=[\frac{\left(\mathrm{absorbance of the sample}-\mathrm{absorbance of the blank}\right)}{\left(\mathrm{absorbance of the control}-\mathrm{absorbance of the blank}\right)}]\times 100$

### RNA isolation, cDNA synthesis and quantitative reverse transcription-polymerase chain reaction 

Total RNA isolation from cultured cells was performed by the AccuZol™ total RNA extraction solution (Bioneer, Korea) according to manufacturer’s instructions. RNA concentration was analyzed by Nanodrop 2000c spectrophotometer (Thermo Scientific, USA). Alterations in mRNA expression of the mentioned genes were evaluated by quantitative reverse transcriptase polymerase chain reaction (qRT-PCR) after 4 hours treatment of the cancer cells with certain percentages (v/v) of the culture supernatants. Reverse transcription of 1 μg RNA from each sample was performed with the PrimeScript RT reagent kit (Takara Bio, Japan). The experiments were implemented in a rotor gene 3000 corbett (QIAGEN Valencia, USA) detection system using SYBR Premix Ex Taq (Takara Bio, Japan). The primer sequences are provided in the Table 1. PCR condition was performed as follow: 

a primary denaturation at 95˚C for 1 minute, and 40 cycles at 95˚C for 15 seconds and 65˚C for 1 minute. Final master mix of the PCR reaction consisted of 10 ml SYBR Green master mix, 2 ml cDNA, 0.5 ml of each forward and reverse primer (10 pmol) and 7 ml nuclease-free water. Experiments were carried out in duplicate for each data point. *Beta-2-microglobulin (B2M)* mRNA was amplified as a normalizer to show relative fold changes in each target mRNA expression. Melting curve analysis was performed to confirm specific PCR product of each primer pair. The effects of LRS and LCS on gene expressions were compared with MRS and MRL. 

### Statistical analysis

Total expression ratio of the genes was compared between treated and control cells using a randomization test applied in the relative expression software tool (REST^©^). 

Mann-Whitney test was used for comparison of pretreated controls with inhibitory concentration 50% (IC_50_) of cells treated with lactobacilli culture supernatants as well as pH and lactate-adjusted as well as pretreated controls in SPSS software (version 16.0). All data were expressed as a mean ± SE of three separate experiments. P<0.05 was considered as statistically significant. 

## Results

### The effects of L. crispatus and L. rhamnosus supernatants on MDA-MB-231 and MRC5 cell proliferation

**Fig.1:**
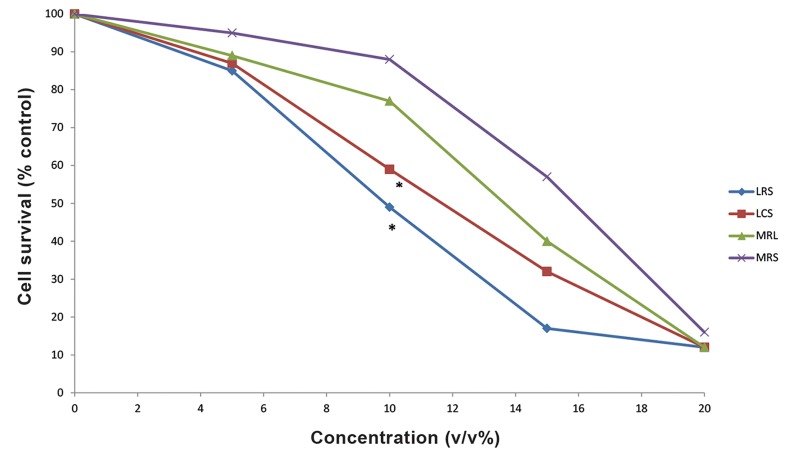
Cell growth inhibitory effect of LCS, LRS, MRS or MRL diffèrent concentrations on the MDA-MB-231 cells. LCS; *Lactobacillus crispatus* culture supernatant, LRS; *Lactobacillus rhamnosus* culture supernatant, MRS ; deMan Rogosa Sharpe and MRL; MRS adjusted with lactate.

LCS and LRS had no toxic effect on MRC5 cells (data not shown). The IC_50_ values of LRS and LCS against MDA-MB-231 cells were 10% (v/v) and 13% (v/v), respectively. The cytotoxic effects of LCS and LRS against MDA-MB-231 cells were higher than MRS and MRL (MRS with pH adjusted to that of LCS and LRS, P<0.05, Fig.1). These results imply that the main cause of cancer cell death was not the acidity, but it can be attributed to a substance other than lactate in the supernatant of the lactobacilli. In addition, cytotoxicity effect of LRS was significantly higher than LCS in MDA-MB-231 (P<0.01).

**Table 1:** Sequence of the primers used in this study


Gene	Sequence (5´-3´)	Product size

Β2M	F:AGATGAGTATGCCTGCCGTG	105 bp
	R: GCGGCATCTTCAAACCTCCA	
SHARP1	F:AACAGCAGTTGAACATGGACG	150 bp
	R: TGTAGGTATCCTTGGTGTCGT	
HIF-1α	F:GGACAAGTCACCACAGGACA	168 bp
	R: GGAGAAAATCAAGTCGTGCTG	
SLC2A1	F:AAGAGAGTCGGCAGATGATG	140 bp
	R: ATAGAAGACAGCGTTGATGC	
XBP1	F:TCTTCAGCAACCAGGGCATC	150 bp
	R: TGAGCGGGAACAGCAAGTG	
VHL	F:CGGACAGCCTATTTTTGCCA	132 bp
	R: TCTTCGTAGAGCGACCTGAC	
HSP90	F:AGTTGAAAAGGTGGTTGTGTCA	158 bp


### Effects of L. crispatus and L. rhamnosus supernatants on the expression of HIF pathway genes

**Fig.2:**
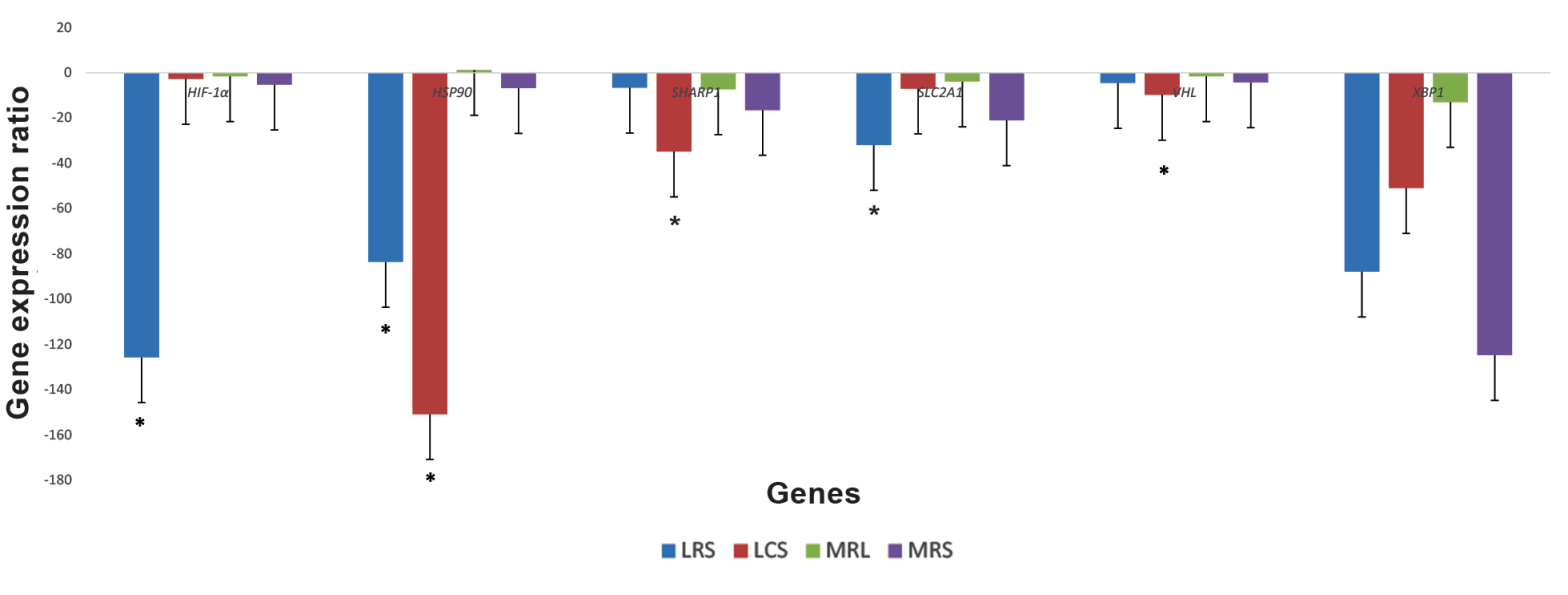
The effects of LCS, LRS, MRS and MRL on expression of the selected genes in the HIF pathway of the MDA-MB-231 cells. LCS; *Lactobacillus crispatus* culture supernatant, LRS; *Lactobacillus rhamnosus* culture supernatant, MRS ; deMan Rogosa Sharpe, MRL; MRS adjusted with lactate and HIF; Hypoxia-inducible factor.

Findings demonstrated that all of the evaluated genes have been expressed in the MDA-MB-231 cell line before treatment. Figure 2 implicates the expression of these genes after treatment with LCS, LRS, MRS and MRL. In this experiment, LRS treatment led to decreased levels of *HIF-1α, HSP90* and *SLC2A1* mRNA expression by the factors 125, 83 and 31, respectively; while LCS treatment down-regulated the expression levels of *HSP90, SHARP1* and *VHL* by the factors 150, 34 and 9, respectively. 

## Discussion

Although it is believed that lactic acid, produced by cancer cells, plays a pivotal role in the development of malignancies (21) and HIF-1 activation, as well as triggering tumor growth and angiogenesis (22), producing this bacterial organic compound has been considered to exert anticancer effects for a long time. However, the exact mechanism of cytotoxic effects of lactobacilli on cancer cells is not obvious yet. Studies have shown that the effect of lactobacilli is different in malignant compared to the normal cells with the same origin (23). Consistent with such studies, we demonstrated that the presented lactobacilli (L. crispatus and L. rhamnosus) supernatants have cytotoxic effects on cancer, but not on normal fibroblast cells. 

Recently, several putative mechanisms have been proposed for anti-cancer effects of lactobacilli, including decrease in the expression of oncogenes as well as modulation of immune system (24). In the present study, in order to find the underlying mechanism of lactobacilli anti-cancer effects, we analyzed the expression of HIF pathway genes and demonstrated that LRS could dramatically down-regulate *HIF-1α* mRNA expression in the MDA-MB-231 cell line. Wang et al. (25) have previously shown that LGG supplementation restored the HIF-2α protein levels, but not HIF-1α in an animal model of alcoholic liver disease, however, the effect of lactobacilli on HIF expression in cancer cells has not been evaluated before. Semenza (16) have shown that treatment of tumorbearing mice with digoxin to reduce HIF-1α expression significantly diminished primary tumor growth and lymph node metastasis, implying that induction of a HIF inhibitor to the therapeutic regimen of the selected group of breast cancer patients can be beneficial. Consequently, in addition to the fact that lactobacilli are considered as probiotics, our present findings, regarding the HIF-1α downregulation due to the LRS treatment, suggest that L. rhamnosus might be served as a HIF inhibitor in therapeutic regimens. 

Furthermore, Laudański et al. (13) demonstrated higher expression level of *SLC2A1* in the MDAMB-231 compared to the MCF-7 cells. Thus, they suggested a putative role for *SLC2A1* in invasiveness of cancer cells. In present study, we showed that LRS treatment down-regulated *SLC2A1* expression level, implicating the therapeutic value of *L. rhamnosus.* Interestingly, the result of the previous investigations, indicating that inhibition of glycolysis might serve a future modality for breast cancer therapy (26), provides further supports for application of lactobacilli in this regard. 

Besides, our study showed significant downregulation of *HSP90* expression following LCS and LRS treatments. Hsp90 is an important element of the chaperone protein family which has been shown to participate in stabilization, regulation, and preservation of oncogenic client proteins, in association with co-chaperones. As a result, Hsp90 and its co-chaperones have been considered as major therapeutic targets for cancer treatment. Various chemical compounds have been tested for their inhibitory effects on Hsp90 with considerable results in some clinical trials (27). Consequently, our results regarding inhibitory effects of LCS and LRS on *HSP90* expression would open a new window for researchers to find new therapeutic *HSP90* inhibitors. 

Although *XBP1* expression has been shown to be decreased following both LRS and LCS treatments, this down-regulation was not significant compared to MRS and MRL. 

In addition, we have demonstrated down-regulation of *VHL* and *SHARP1* tumor suppressor gene expressions by LCS treatment but not LRS, which implies that LRS would be a better choice for HIF pathway specific treatment modalities. The higher cytotoxic effects of LRS than LCS in breast cancer cells also supports this idea. However, since culture supernatants of these bacteria may contain various fractions with possible synergistic as well as antagonistic effects on gene expressions, further analyses could help to find the foremost fraction required for each purpose. Although we demonstrated the cytotoxic effects of both LCS and LRS on the MDA-MB-231 cells, it is proposed that such effect of LRS could not be attributed to HIF pathway regulation. Thus, further investigations are required to determine the potential effect of LRS on the other molecular pathways involved in tumorigenesis. None the less, it is suggested that LRS effect on the MDA-MB-231 cells can at least be partially due to down-regulation of HIF-1, as a master regulator of O^2^ homeostasis (28) and a factor facilitating breast cancer metastatic niche (29). With regards to the role of HIF-1α overexpression in inducing breast cancer related germ-line mutation (30), in one hand, and the role of lactobacilli in inhibiting activity of this pathway, on the other hand, evaluating lactobacilli effects on hereditary breast cancer could help to better perception of the mechanisms contributing to this abnormality and consequently finding a potential therapeutic approach. 

## Conclusion

Both LCS and LRS have cytotoxic effects on the MDA-MB-231 cells, although the LRS effects on these cells are more prominent. LRS has shown to down-regulate the expression of some oncogenic targets, with no effect on tumor suppressor genes, in HIF pathway. Consequently, LRS would be an appropriate candidate for HIF pathway specific inhibition modalities. As inhibition of HIF pathway suggests a promising strategy in cancer treatment, our findings could help to find a novel therapeutic approach for this disease. However, further investigations are needed to evaluate expression of other target genes in this pathway, following LRS treatment. 
